# Mr. Lister

**Published:** 1881-02

**Authors:** 


MR. LISTER.—The Royal Medal of the Royal Society has been conferred on Professor Lister, in recognition of his important physiological services, and the advance in surgery due to his studies and application of antiseptic principles.

Mr. Lister is spoken of as the next President of the Clinical Society of London.—*Medical News.*

